# The High-benefit Approach: A New Targeting Strategy in Precision Medicine

**DOI:** 10.31662/jmaj.2025-0162

**Published:** 2025-09-26

**Authors:** Kosuke Inoue

**Affiliations:** 1 Department of Social Epidemiology, Graduate School of Medicine, Kyoto University, Kyoto, Japan; 2 Hakubi Center, Kyoto University, Kyoto, Japan

**Keywords:** heterogeneity, precision medicine, high-benefit approach, causal inference, machine learning

## Abstract

In medicine, treatment or intervention is typically prioritized for individuals at high risk of diseases or mortality. This high-risk approach, which focuses on “high-risk” patients, has been a cornerstone of clinical decision-making. Additionally, recent advancements in precision medicine, especially those using multi-omics data to assess disease risk, have frequently been framed within this approach. However, it is not always the case that individuals at high risk of disease benefit most from the treatment of interest. In this context, we previously proposed a novel targeting strategy called the “high-benefit approach.” By applying machine learning technologies to data from randomized controlled trials or observational studies, this strategy allows us to identify subpopulations likely to experience substantial benefits from the treatment and to target them, rather than those who are simply at high risk of diseases. The high-benefit approach contributes not only to effective resource allocation but also to the potential mitigation of health disparities by identifying individuals with limited or no benefits and offering alternative approaches that would be effective for them. This review paper explains the overall concept of the high-benefit approach and its potential for application in healthcare literature, particularly in advancing future precision medicine and public health.

## Introduction

In 1984, Geoffrey Rose outlined two public health approaches, the population approach and the high-risk approach, and clarified their fundamental differences ^(1)^. The population approach aims to shift the entire distribution of risk factors across a population. This approach emphasizes societal or regulatory changes that affect everyone, such as tobacco control, food industry regulations, or vaccination, rather than relying solely on individual interventions. It is considered potentially transformative because it establishes new social norms that make healthy choices easier for the entire population. Furthermore, it can particularly benefit hard-to-reach groups; however, the benefits for individuals may be limited, and its effectiveness depends largely on adherence and continued support from society or governing bodies for mass strategies.

The high-risk approach targets individuals who are at high risk of developing specific health conditions, making it more personalized than the population approach. Because clinicians focus on individual care, such as diagnosis, prescribing medication, and performing surgery, this approach is commonly applied in clinical settings. However, it is important to note that people at high risk are not always the ones who benefit from treatment. Continuing treatment for high-risk individuals who do not respond may fail to improve their health outcomes and may even cause harm due to side effects. Moreover, the use of arbitrary risk thresholds (e.g., a 10-year cardiovascular risk >20%) may result in withholding treatment from individuals classified as “low risk” who could, in fact, benefit.

To address these issues, we previously proposed “the high-benefit approach” as a new targeting strategy that prioritizes treatment responsiveness over disease risk (i.e., benefit) and targets individuals who are most likely to benefit ^(2)^. In this narrative review, we discuss the overall concept of the high-benefit approach and its potential for application in precision medicine and public health.

## Estimating Benefits from Treatment at the Individual Level

**Figure 1.**
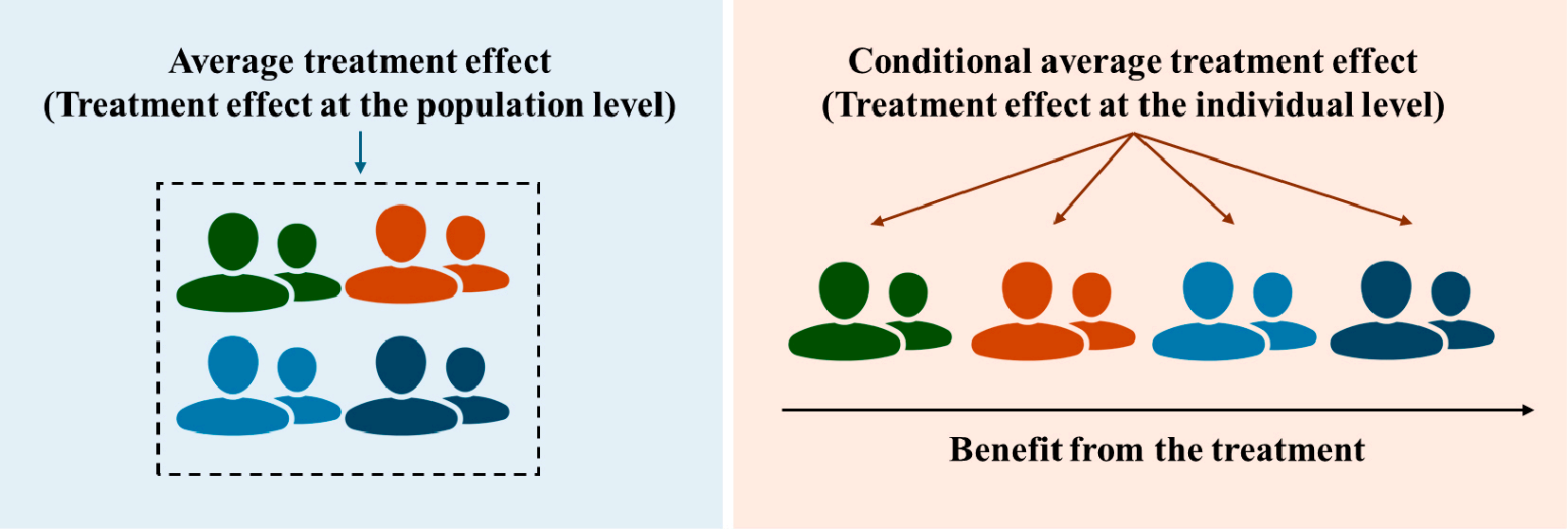
Estimating the treatment effect at the individual level.

The most important aspect of the high-benefit approach is the estimation of the benefit (or harm) from treatment for each individual (Figure 1). Although the actual treatment effect at an individual level cannot be observed, because each individual can receive only one type of treatment strategy, it can be estimated under the counterfactual framework with several causal assumptions (e.g., conditional exchangeability, positivity, consistency, etc.). More formally, the average treatment effect (ATE) represents the treatment effect among the entire population (*E* [*Y_a_*−Y_a_*)]), where *Y* represents the outcome, *A* represents the treatment or exposure (*a*, index; a*, reference), and *Y_A=a_* represents the potential outcome if the treatment had taken value *A* = *a*. In addition, the conditional average treatment effect (CATE) represents the treatment effect conditional on a set of observable individual characteristics (*L*) such as age, sex, and race (*E* [*Y_a_*−Y_a_*|*L*]). By estimating CATE, we can assess the treatment benefit or harm for each individual or subgroup of individuals (defined by *L*), and thus assess heterogeneity in the treatment effect across individuals.

## Recent Machine Learning Approaches to Estimate the Benefit from Treatment Across Individuals

Recent advancements in machine learning have introduced robust methodologies for detecting and analyzing treatment effect heterogeneity ^(3)^. These algorithms can uncover complex, non-linear interactions between treatment outcomes and a high-dimensional set of covariates―patterns that traditional statistical approaches often fail to detect. This approach enhances the analysis of both randomized controlled trials and observational data, enabling researchers to estimate treatment effects based on individual characteristics and ultimately to identify optimal treatment strategies.

**Figure 2.**
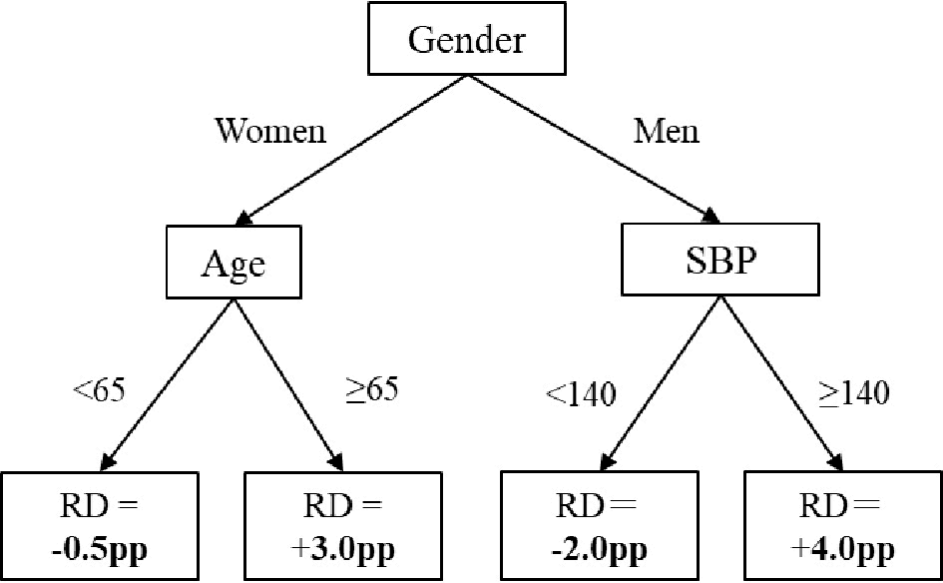
A hypothetical example of a causal tree. Pp: percentage point; RD: risk difference; SBP: systolic blood pressure. This example shows only three variables in the tree, but actual trees are more complex with several variables and splits.

The causal forest algorithm is one of the most commonly used machine learning algorithms for estimating treatment effect heterogeneity in the healthcare literature ^(3)^. This method employs tree-based algorithms that synthesize the outputs from various decision (causal) trees into a prediction model for the treatment effect ^(4), (5), (6)^. Each tree systematically groups samples based on distinct characteristics to enhance the predictive accuracy of the treatment effect. For instance, Figure 2 illustrates an example of a causal tree in which the treatment decreases adverse events by 3.0 percentage points among women older than 65 years, while potentially increasing adverse events by 2.0 percentage points in men with a systolic blood pressure below 140 mmHg. Although this is a highly simplified example, in actual applications, we include many covariates (e.g., age, gender, socioeconomic status, health measurements, comorbidities, and medications) to build thousands of trees and generate the final output.

The causal forest algorithm is different from other commonly used machine learning algorithms for outcome prediction in healthcare research, such as the random forest algorithm. In brief, the causal forest algorithm is specifically designed to estimate how much a treatment would provide benefit or harm (i.e., treatment effect) for each patient based on their individual characteristics, while the random forest algorithm is designed to predict outcomes themselves (i.e., the risk of disease) based on individual characteristics. In recent years, many other methods have also been developed to estimate the treatment effect at the individual level, and details of these methodologies can be found in prior literature ^(3), (7)^.

## Application Examples

In this section, I present several application examples of the high-benefit approach.

### Who should be targeted for intensive blood pressure control?

The first application example is from a clinical setting using the Systolic Blood Pressure Intervention Trial (SPRINT) ^(8)^. The SPRINT is a large randomized controlled trial in which the authors reported that intensive blood pressure management could reduce cardiovascular disease risks by 25% compared to standard approaches. The original analysis did not reveal significant treatment effect differences across demographic groups. However, in a post-hoc analysis, we identified that the effectiveness of intensive blood pressure control varied among Black individuals based on whether they were living alone or with others ^(9)^. This finding of heterogeneity by race and living arrangement highlights the importance of simultaneously considering the complex interplay of multiple characteristics when assessing who benefits most from intensive blood pressure control. Therefore, we applied causal forest algorithms to the SPRINT data to examine the heterogeneity in the cardioprotective effect of intensive blood pressure control ^(2)^.

**Figure 3.**
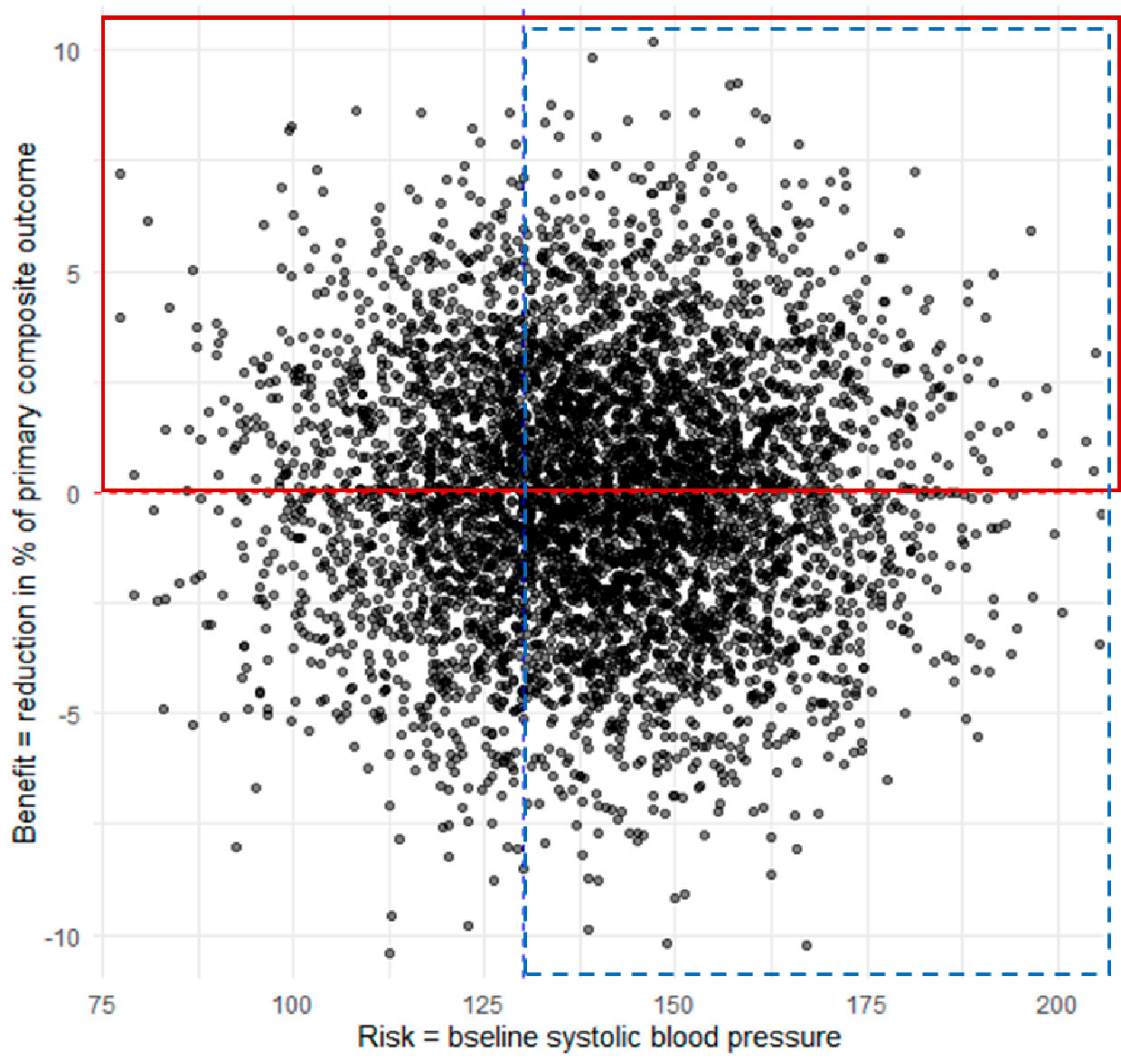
High-benefit approach vs. high-risk approach. The data was simulated based on the findings from SPRINT. The traditional high-risk approach targets individuals in the blue dashed box, while the high-benefit approach targets individuals in the red solid box. For the scatter plot in the original study, please see Inoue et al.^(2)^ SPRINT: Systolic Blood Pressure Intervention Trial.

Figure 3 shows baseline systolic blood pressure on the horizontal axis and the CATE of intensive blood pressure management on the vertical axis. The traditional high-risk approach, marked with blue dotted lines, targets individuals with systolic pressures of 130 mmHg or higher. In contrast, the high-benefit approach, marked with red lines, targets those who are expected to achieve a reduction in cardiovascular disease risk, with a focus on effect sizes from zero upward. When these strategies were compared, the high-benefit approach was shown to yield a treatment effect (risk difference) that is five times greater than that achieved when using the high-risk approach (high-benefit approach, +9.36 [95% confidence interval [CI]: 8.33-10.44] vs. high-risk approach, +1.65 [95% CI: 0.36-2.84] percentage points; p < 0.001) ^(2)^. The results were consistent when using the cardiovascular risk score at baseline instead of blood pressure as a risk marker. These findings indicate the potential of the high-benefit approach to maximize the effectiveness of intensive blood pressure control in future precision medicine.

### Who benefits from health insurance coverage for blood pressure management?

The assessment of treatment effect heterogeneity is also increasingly recognized in policy evaluation research. Here, we present findings from two studies based on the Oregon Health Insurance Experiment ^(10)^, which evaluated the effects of Medicaid, a public health insurance program in the United States, on low-income residents of Oregon. In the original study, researchers found that while Medicaid significantly improved access to healthcare and provided financial protection, its effects on physical health measures were less clear. In particular, Medicaid coverage was associated with substantial improvements in mental health but showed no significant impact on physical health indicators such as blood pressure ^(10)^. To better understand these mixed findings, especially the lack of significant improvement in blood pressure, we aimed to examine whether the overall results might have masked meaningful benefits in specific subgroups. Using causal forest and instrumental variable methods, we analyzed the heterogeneous treatment effects of Medicaid coverage on both blood pressure and mental health outcomes.

By applying causal forest, we found that a certain subgroup of individuals experienced improved blood pressure as a result of Medicaid coverage, while such benefits were obscured by others who did not benefit from the insurance ^(11)^. Of interest, the high-benefit group comprised individuals with minimal or no prior healthcare expenditures before the intervention, highlighting that Medicaid significantly supports those who were previously underserved by healthcare systems at baseline. We also identified that the effect of Medicaid coverage on mental health was heterogeneous, showing that older individuals with extensive physical or mental health conditions at baseline were most likely to experience the largest reduction in depression risks due to Medicaid coverage ^(12)^. These findings emphasize the value of examining heterogeneity in treatment effects across subgroups―even when targeted interventions are not possible, as with statewide policies―because doing so helps us to understand the underlying mechanisms of overall findings and to design better strategies to maximize population health in society ^(13)^.

## Identifying Interpretable High-benefit Subpopulations

Data-driven approaches in healthcare present a paradox in which the inherent flexibility of non-parametric models, although beneficial, often does not align well with practical decision-making. As illustrated above, these models are particularly effective at breaking down the complexity of treatment effect heterogeneity by estimating CATEs as non-linear functions of multiple individual characteristics. In contrast, clinicians typically use simpler, established thresholds in their treatment decisions, such as initiating high-intensity statin therapy in patients with a high cardiovascular risk ^(14)^. Although efforts have been made to identify key characteristics that substantially influence heterogeneity, the challenge remains that thresholds derived from purely data-driven approaches may not always correspond to the practical requirements of users, which is a significant obstacle to the effective application of these models in the real world.

To address these challenges, we recently proposed a new framework called pragmatic subgroup discovery ^(15)^. This framework aims to achieve high predictive accuracy, relevance, and interpretability of the final output model by conducting the following two principal steps: estimating CATE (Step 1) and identifying relevant subgroups (Step 2). In Step 1, CATE is estimated using statistical and machine learning models, such as causal forest, including high-dimensional individual characteristics (e.g., continuous age, continuous blood pressure, etc.) as described above. In Step 2, by building tree-based algorithms, such as the classification and regression tree, to predict CATE, and by carefully selecting and potentially modifying covariates that are critical for decision-making (e.g., age >65 years old, systolic blood pressure >130 mmHg, etc.), we aim to identify interpretable subgroups with heterogeneous treatment effects. This novel framework refines the use of non-parametric effect models, enhancing the credibility of these analyses for heterogeneous treatment effect estimation and improving scientific communication to facilitate the application of the high-benefit approach in real-world settings.

## Applying the High-benefit Approach in Future Precision Medicine

**Figure 4.**
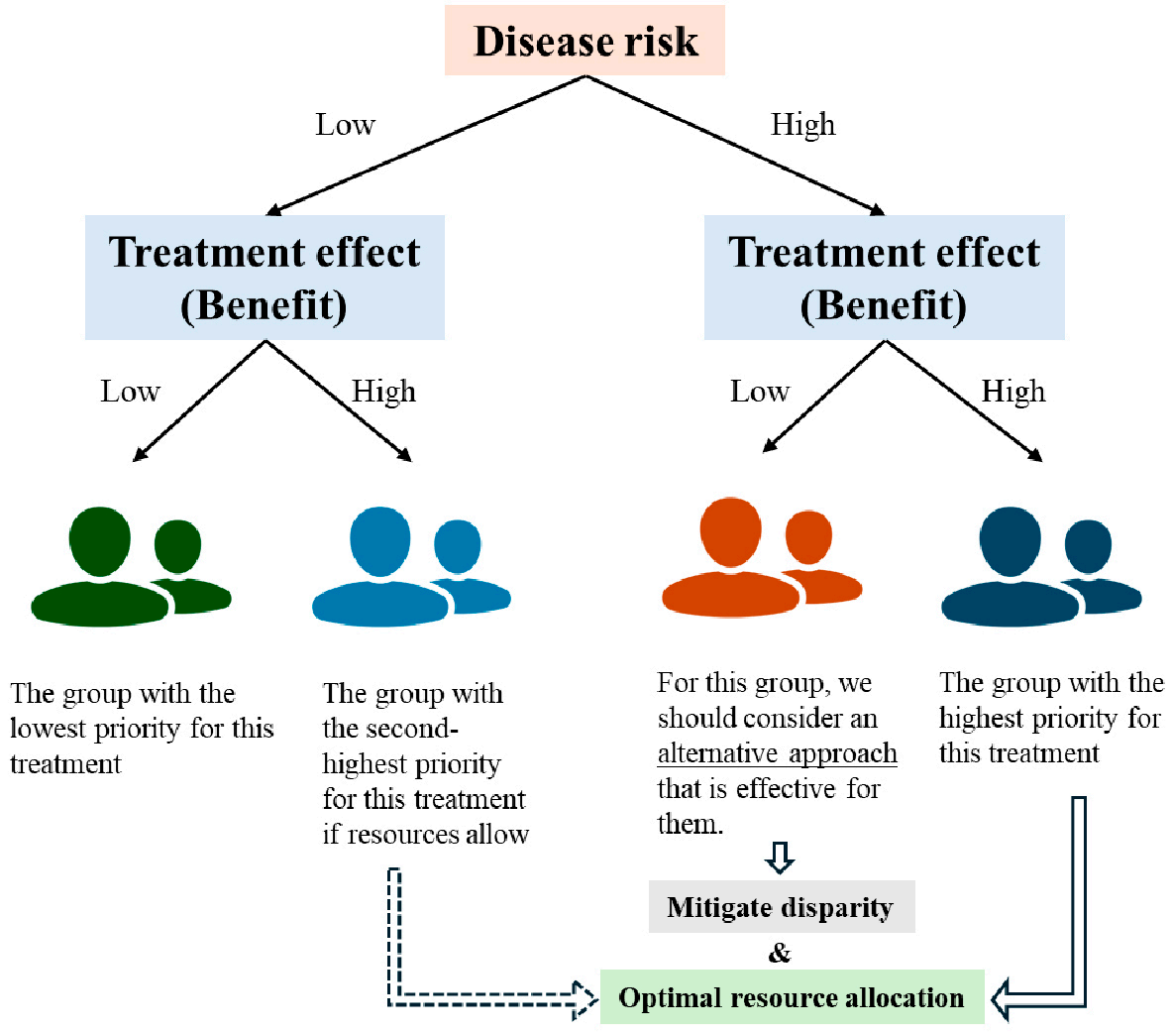
High-benefit approach to optimize resource allocation and mitigate health disparities.

Effective resource allocation in healthcare requires the precise identification of the high-benefit group, as it allows us to prioritize interventions for groups with both high risk and high benefit (Figure 4). In this context, the high-benefit approach would result in a paradigm shift in the targeting strategy (by shifting our focus from risk to benefits) for specific interventions or treatments that are of interest in future precision medicine and public health.

For example, coronary computed tomography (CT) screening is useful for detecting coronary artery calcium, a major marker of subclinical cardiovascular disease ^(16)^; however, it is impractical to screen the entire population using this method. Although current clinical guidelines primarily target high-risk individuals, they may overlook those at low risk who could still benefit. Indeed, in our previous study using the Multi-Ethnic Study of Atherosclerosis cohort, we found that a substantial number of individuals with low predicted cardiovascular risk had a high burden of coronary artery calcium, suggesting they may also benefit from coronary CT screening, while they are not eligible under the current high-risk approach ^(17)^.

Moreover, given the crucial role of genetic information in precision medicine, it is also important to consider whether individuals with a high genetic predisposition to certain diseases derive greater benefits from preventive strategies. Using the biobank cohort in the UK and Japan, we explored the heterogeneity in cardiometabolic disease risk associated with lifestyle factors, such as obesity and smoking, and its correlation with genetic risk for cardiometabolic diseases ^(18)^. Of note, although we found heterogeneity in disease risk associated with obesity or smoking across the high-dimensional combination of individual characteristics, a weak or negative correlation with the polygenic risk score was mostly observed. This suggests that genetically high-risk groups will not necessarily benefit the most from preventive measures against obesity and smoking. These results highlight the need to consider not only disease risk but also treatment effectiveness to maximize the overall population benefit from treatment or intervention with limited resources.

## Applying the High-benefit Approach to Address Health Disparities

Another important point to consider is whether targeting specific individuals can mitigate or exacerbate existing health disparities (Figure 4). For groups characterized by high risk but low benefit from the treatment, the presence of a treatment effect on average does not necessarily translate into individual benefits (because of “low benefit”). This issue is particularly relevant to populations with high social risks, such as those with low incomes or education levels, where not accounting for treatment effect heterogeneity could exacerbate health disparities under “evidence-based” practices ^(19)^. Identifying these groups to explore alternative treatments and mechanisms is critical to mitigate such disparities. Moreover, adapting treatment approaches from multiple options (e.g., selecting the most effective prescription among sodium-glucose transporter 2 inhibitors, glucagon-like peptide-1 receptor agonists, and biguanides based on the estimated treatment effect in each individual in diabetes care) allows us to provide the best treatment strategy for each individual, thus potentially mitigating the existing health disparity. The effectiveness of social health interventions, whether through traditional media (e.g., flyers) or digital platforms (e.g., apps), also varies across individual (time-fixed and time-varying) characteristics. Rigorous evaluation of these strategies, accounting for such heterogeneity, is essential for effective and equitable implementation of personalized approaches for a better society.

### Conclusion

Although medicine traditionally prioritizes high-risk individuals, our “high-benefit approach” has the potential to effectively and efficiently improve population health by optimizing the allocation of medical resources. By clarifying individuals who do not benefit (or even be harmed) from treatment and considering alternative strategies for them, this approach can also help mitigate existing health disparities. With recent advancements in data availability and data science, we believe the high-benefit approach will play an important role as a third strategy, in addition to the population approach and the high-risk approach, in future precision medicine and public health.

## Article Information

This article is based on the study, which received the Medical Research Encouragement Prize of The Japan Medical Association in 2023.

### Conflicts of Interest

Kosuke Inoue receives research support or grants from the Japan Medical Association, the Japan Society for the Promotion of Science (23KK0240, 25K02887), the Japan Science and Technology (JST PRESTO; JPMJPR23R2), and the Japan Agency for Medical Research and Development (AMED; JP22rea522107). No other disclosures were reported.
